# MTA1 as negative prognostic marker in vulvar carcinoma

**DOI:** 10.1007/s00432-023-04579-4

**Published:** 2023-01-23

**Authors:** Giulia Wanka, Julia Jueckstock, Carl Mathis Wild, Aurelia Vattai, Sophie Fürst, Helene H. Heidegger, Christina Kuhn, Elisa Schmoeckel, Udo Jeschke, Christian Dannecker

**Affiliations:** 1 Department of Obstetrics and Gynecology, University Hospital Augsburg, Stenglinstraße 2, 86156 Augsburg, Germany; 2 Department of Obstetrics and Gynecology, University Hospital, LMU Munich, Marchioninistraße 15, 81377 Munich, Germany; 3 Department of Pathology, LMU Munich, Thalkirchner Straße 142, 80337 Munich, Germany; 4 Department of Obstetrics and Gynecology, RoMed Clinic, Krankenhausstraße 2, 83512 Wasserburg am Inn, Germany

**Keywords:** Vulvar carcinoma, MTA1, Clinicopathological factors, Metastasis, Cancer survival, Prognosis

## Abstract

**Purpose:**

Vulvar cancer is the fourth most common malignancy of the female genital tract after endometrial, ovarian, and cervical carcinoma and affects mainly elderly women. In 2020 there were registered more than 17,000 deaths worldwide related to vulvar carcinoma. Data about target-based therapies and predictive biomarkers for vulva carcinomas are rare so far. The metastasis-associated gene MTA1 is a transcriptional repressor with a potential effect on cancer. Expression of MTA1 was found to be significantly enhanced in gynecological malignancies as breast or ovarian cancer tissues with advanced cancer stages and higher FIGO grading, indicating an important role of MTA1 in the progression of those tumor entities. Due to the lack of information around MTA1 and its significance regarding vulvar carcinoma, this study focuses on the expression of MTA1 in vulvar carcinoma and its correlation to clinicopathological characteristics and prognosis.

**Methods:**

A total of 157 paraffin-embedded vulvar cancer tissues were immunohistochemically stained and examined for MTA1 expression by using the immunoreactive score. Subsequently, the values were correlated with clinicopathological parameters.

**Results:**

MTA1 was found to be expressed in 94% of the patients in the cytoplasm and 91% in the nucleus. Cytoplasmatic expression of MTA1 was significantly increased in non-keratinizing squamous cell carcinoma and in vulvar carcinoma of the condylomatous type, compared to keratinizing squamous cell carcinoma and vulvar carcinoma of the verrucous type. High MTA1 expression in the nucleus was associated with advanced tumor size as well as higher FIGO grading. In addition, p16 negative vulvar carcinomas showed a higher nuclear expression of MTA1 compared to p16 positive vulvar carcinomas. Suprisingly, Kaplan–Meier analysis showed a significantly lower disease-free survival in tumor samples without a nuclear expression of MTA1.

**Conclusions:**

MTA1 was identified as a negative prognostic marker for vulvar carcinoma associated with advanced tumor stage and FIGO grading. A possible explanation could be that the antibody used for this study does not bind to a possible mutation in the C terminal region of MTA leading to negative immunohistochemical staining and this can be correlated with early recurrence in patients with vulvar carcinoma.

## Introduction

Vulvar carcinoma is considered a rare female malignancy, making up about 5% of all gynecologic cancers, and affecting mainly elderly women (Miller et al. 2016). However, over the past few decades, the incidence of vulvar cancer and vulvar intraepithelial neoplasia (VIN) has reportedly increased, particularly among younger women (Joura et al. 2000; Judson et al. 2006). The most common subtype is squamous cell carcinoma (VSCC). One type of VSCC, accounting for approximately 80%, is associated with lichen sclerosus (LS) and/or differentiated vulvar intraepithelial neoplasia (dVIN) as premalignant lesions and is more prevalent in older patients. The remaining 20% of VSCC, is human papillomavirus (HPV)-driven and affects mostly younger women in the fourth to sixth decade. The associated premalignancy is called VIN of the usual type (uVIN) (Hoang et al. 2016; Faber et al. 2017). In addition to keratinizing and non-keratinizing VSCC other morphological variants have been described including basaloid and verrucous carcinoma. Keratinizing variants are considered HPV-negative, whereas basaloid and verrucous squamous cell carcinomas are more likely to be related to human papillomavirus (Hoang et al. 2016). Data describe a less favorable outcome in HPV-independent vulvar carcinoma in contrast to HPV-dependent vulvar carcinoma (Singh and Gilks 2020). Beside HPV, the development of vulvar carcinomas is associated with other risk factors as immunosuppression, smoking, chronic inflammatory dermatosis as lichen sclerosus and metasynchronous genital lesions (Satmary et al. 2018)*.* Tumor size and spread are classified by the TNM classification as well as the FIGO grading system visualized in Table 1. Surgical intervention represents the primary treatment modality for locally resectable vulvar cancer. Often the prognosis is limited by lymph node invasion and high recurrence rates (Mahner et al. 2015; Te Grootenhuis et al. 2016). Medicamentous therapy approaches are mainly used in palliative settings or in cases of recurrence (Tomao et al. 2012). Based on mentioned data, target-orientated therapy strategies addressing specific tumorigenic pathways need to be investigated to improve the clinical outcome of patients.

**Table 1:** Clinicopathological characteristics of the analyzed patients’ collective

Clinicopathological parameters		*n*	Percentage (%)
Histology	Keratinizing VSCC	160	90.4
	Warty/basaloid VSCC	17	9.6
Tumor size	T1	69	39
	T2	92	52
	T3	9	5.1
	missing	7	3.9
Nodal status	N0	78	44.1
	N	38	21.5
	N	12	6.8
	missing	49	27.6
Metastasis	M0	8	4.5
	missing	169	95.5
FIGO score	I	61	34.4
	II	54	30.5
	III	47	26.6
	IV	9	5.1
	missing	6	3.4
Grading	G1	29	16.4
	G2	108	61
	G3	39	22
	missing	1	0.6
p16 status	Positive	38	21.5
	Negative	57	32.3
	missing	82	46.3

The protein MTA1 has already been investigated as a potential prognostic factor in other gynecologic tumors, such as ovarian, cervical, and endometrial carcinoma, and correlated with tumor progression and metastases (Balasenthil et al. 2006; Dannenmann et al. 2008; Bruning et al. 2014). Comparable studies do not exist to date in patients with vulvar carcinoma. MTA1 was primarily identified and linked to metastasis by using a rat mammary carcinoma cell line. It was observed that cell lines with a high metastasis rate showed an overexpression of MTA1 mRNA compared to cell lines with a low metastasis rate (Toh et al. 1994). MTA1 is a component of the nucleosome remodeling and histone deacetylation (NuRD) complex (Xue et al. 1998). The NuRD complex is involved in the mechanism of chromatin remodeling. In this process, histon deacetylases remove an acetyl group from the histone leading to chromatin compaction and thereby resulting in the repression of transcription (Toh and Nicolson 2009). Beside transcriptional silencing of target genes MTA1 plays an important role in other mechanisms facilitating tumor metastasis and invasion, including loss of the cell adhesion molecule E-cadherin (Schmalhofer et al. 2009) and epithelial-mesenchymal transition (EMT) (Meng and Wu 2012). EMT describes the ability of tumor cells to disseminate via blood and lymphatic pathways by leaving the tumor origin and forming metastases via reversed mesenchymal-epithelial transition (Wong and Hynes 2006). The mechanism of EMT is of particular relevance in vulvar carcinoma since more than 95% of all vulvar carcinomas are of epithelial origin. Since no information about the expression and involvement of MTA1 in vulvar cancer exists to date, we analyzed the expression of MTA1 in tissue samples and its relation to the clinicopathological parameters of the study group.

## Methods

### Patients collective

A total of 177 patients with vulvar carcinoma, primarily diagnosed between 1990 and 2008 and treated at the department of Gynecology and Obstetrics of the Ludwig-Maximilians-University in Munich, Germany was included in this study. Tissue samples were surgically obtained and histologically processed as part of routine clinical care. For immunohistochemical staining, 157 of the 177 samples were available. Patients’ clinical data were collected, and follow-up examinations were carried out. The collective is the same as described by Wanka et al. in previous studies by our research group (Wanka et al. 2020).

### Immunohistochemistry

To perform immunohistochemical staining formalin-fixed paraffin-embedded (FFPE) tissues were cut by microtome to 3 µm slides and mounted on SuperFrost Plus microscope slides (Menzel Glaeser, Braunschweig, Germany). Tissue specimens were deparaffinized in xylol for 20 min (min), followed by washing in 100% ethanol. To stop the activity of endogenous peroxidase the slides were incubated with 3% hydrogen peroxide diluted in methanol for 20 min before rehydrating them in a descending alcohol series (100%, 70%, 50%). After rinsing with distilled water, the slides were heated with citric acid buffer in a pressure cooker to unmask the antigens’ epitopes. Subsequently, sections were washed two times with phosphate-buffered saline (PBS). Blocking and antibody staining procedures were performed using Zytochem-Plus HRP Polymer-kit (Zytomed, Berlin, Germany). First blocking solution was applied for 5 min, then samples were incubated with the polyclonal rabbit-IgG anti-MTA1 antibody (Bethyl, Montgomery, USA) diluted at the ratio of 1:100 for 16 h (h) at 4 °C in a humid chamber. Post-block reagent was applied, and the samples were incubated for 20 min at room temperature. HRP-Polymer was added and incubated for 30 min. After each incubation with the primary antibody, post-block and HRP-Polymer the slides were rinsed two times with PBS. The addition of 3,3′-Diaminobenzidine (Dako, Hamburg, Germany) started the peroxidase substrate staining reaction which led to the color precipitation which was detectable with a light microscope. Finally, slides were counterstained with hemalum, dehydrated, and covered with glass. To validate the staining method slides made from placenta tissue were used as a positive control.

Specimens were evaluated under the light microscope (Leitz, Wetzlar, Germany) using the semi-quantitative immunoreactive Score (IRS) by Remmele and Stegner (Remmele and Stegner 1987). Describing the product formed by the two parameters optical staining intensity and percentage of positively stained cells. MTA1 showed two different expression patterns in the evaluation, therefore, its cytoplasmic and nuclear staining were independently evaluated by IRS. Patients’ data were correlated with the IRS itself as well as the two individual constituent parameters of the score.

### Statistical analysis

For statistical analysis, the SPSS Statistics version 25 (IBM Corp., Armonk, NY, USA) was used. Non-parametric Kruskal–Wallis test was used to compare between and among groups. Correlation analyses were performed using the Spearman rank correlation coefficient. Kaplan–Meier curves were generated using collected survival data, and differences were tested by the log-rank test. The level of statistical significance was accepted at *p* ≤ 0.05 and all tests were two-sided.

## Results

### Study group and clinical data

Patients’ median age was 67 years (range 20–96 years). 90% of the patients showed a keratinizing VSCC, the remaining 10% a warty/basaloid VSCC. Follow-up examinations were carried out with a mean follow-up time of 71.4 months (range 0–276.7 months). Overall, 121 out of the 177 patients (68%) died during the follow-up period. Further relevant patient characteristics are displayed in Table 2.

**Table 2:** TNM classification and FIGO staging for carcinoma of the vulva, adapted by (Olawaiye et al. 2021)

TNM	FIGO	Description
T1 N0 M0	I	Tumor confined to the vulva
T2 N0 M0	II	Tumor of any size with extension to lower one-third of the urethra, lower one-third of the vagina, lower one-third of the anus with negative nodes
T1 or T2, N1 or N2, M0	III	Tumor of any size with extension to upper part of adjacent perineal structures, or with any number of nonfixed, nonulcerated lymph node
T1 or T2, N3, M0 or T3, Any N, M0 or Any T, Any N, M1	IV	Tumor of any size fixed to bone, or fixed, ulcerated lymph node metastases, or distant metastases

### MTA1 expression in vulvar cancer tissue

**Fig. 1:**
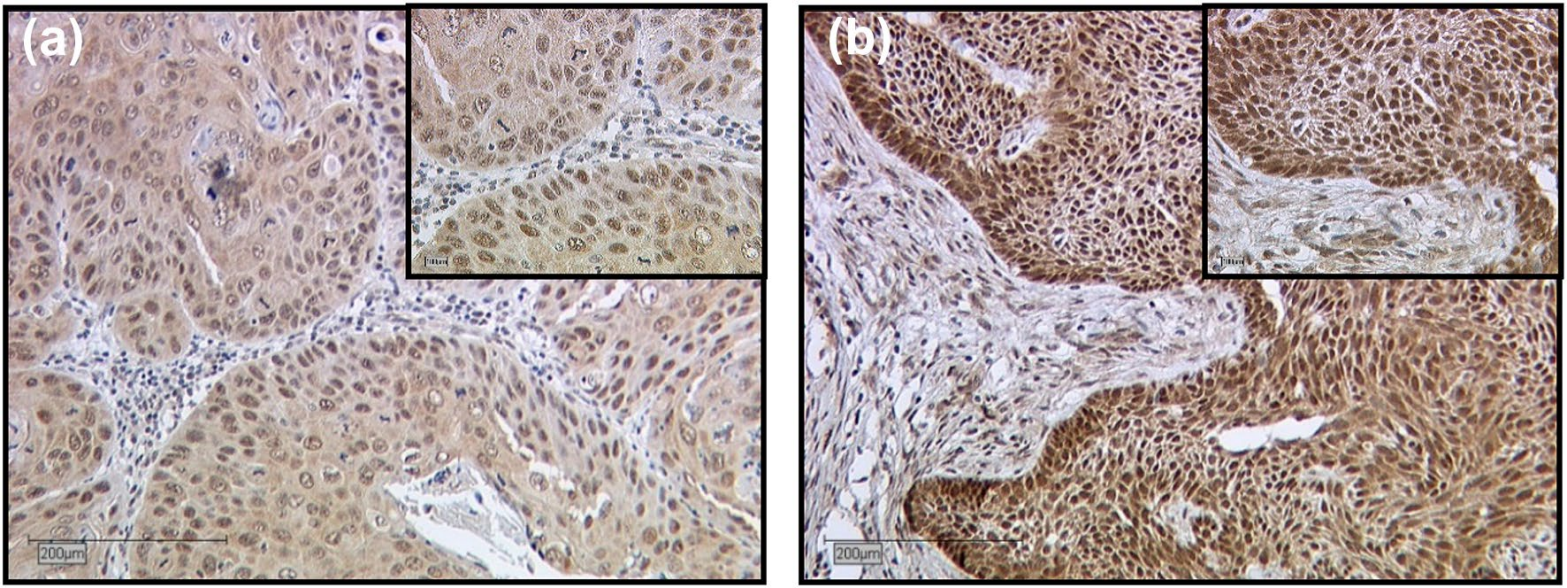
Immunohistochemical staining of MTA1 in vulvar cancer tissue. Representative photomicrographs are presented (10 × and 25 × magnification): **a** intensity score 2; cytoplasmatic and nuclear expression of MTA1 in vulvar carcinoma; **b** intensity score 3; cytoplasmatic and nuclear expression of MTA1 in vulvar carcinoma

The MTA1 staining process in the cytoplasm showed a weak color reaction (IRS 1–2) in 28.6% of the vulvar carcinoma tissue sections, 61.1% showed a moderate color reaction (IRS 3, 4, 6), whereas 4.8% of the specimens showed a strong color reaction (IRS 8, 9, 12) of MTA1. In 5.6% MTA1 was not expressed in the cytoplasm (IRS 0). Nuclear staining of MTA1 resulted in a weak color reaction in 23.8% of the vulvar carcinoma tissue sections (IRS 1–2), 60.3% showed a moderate (IRS 3, 4, 6), and 7.2% a strong color reaction (IRS 8, 12). In 8.7% MTA1 was not expressed in the cell nucleus (IRS 0). Representative photomicrographs are presented in Fig. 1.

**Fig. 2:**
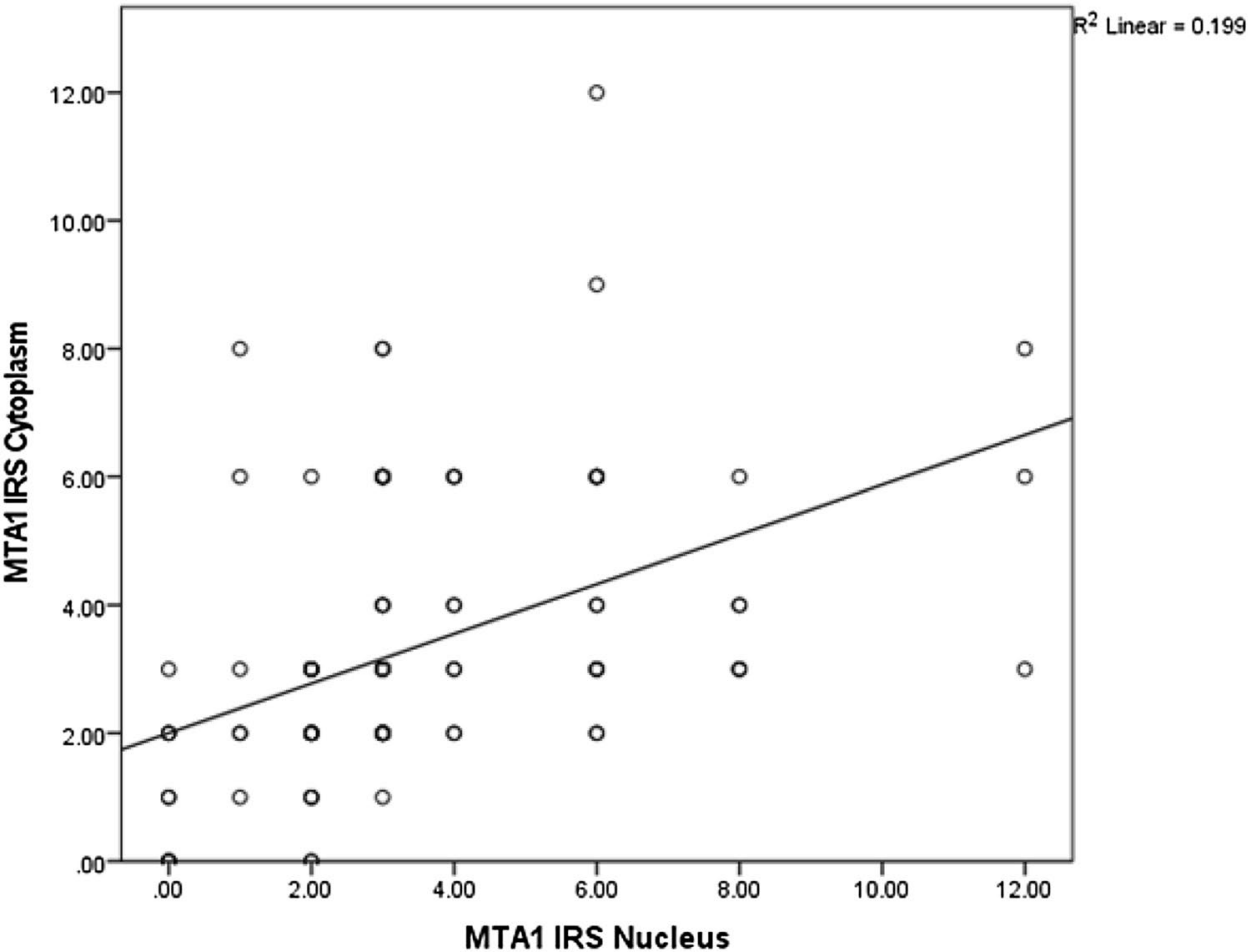
Correlation analysis of cytoplasmatic and nuclear expression of MTA1. Significant correlation between cytoplasmatic and nuclear expression of MTA1 (*ρ* = 0.547, *p* = 0.000–0.001)

The average IRS for the MTA1 expression in the cytoplasm as well as in the cell nucleus was 3.3 (total range 0–12). A correlation analysis using the Spearman correlation function was performed showing a significant positive correlation (*ρ* = 0.547, *p* = 0.000–0.001), indicating that the higher the IRS is in the cytoplasm, the higher it is in the nucleus (Fig. 2).

### Tumor histology

**Fig. 3:**
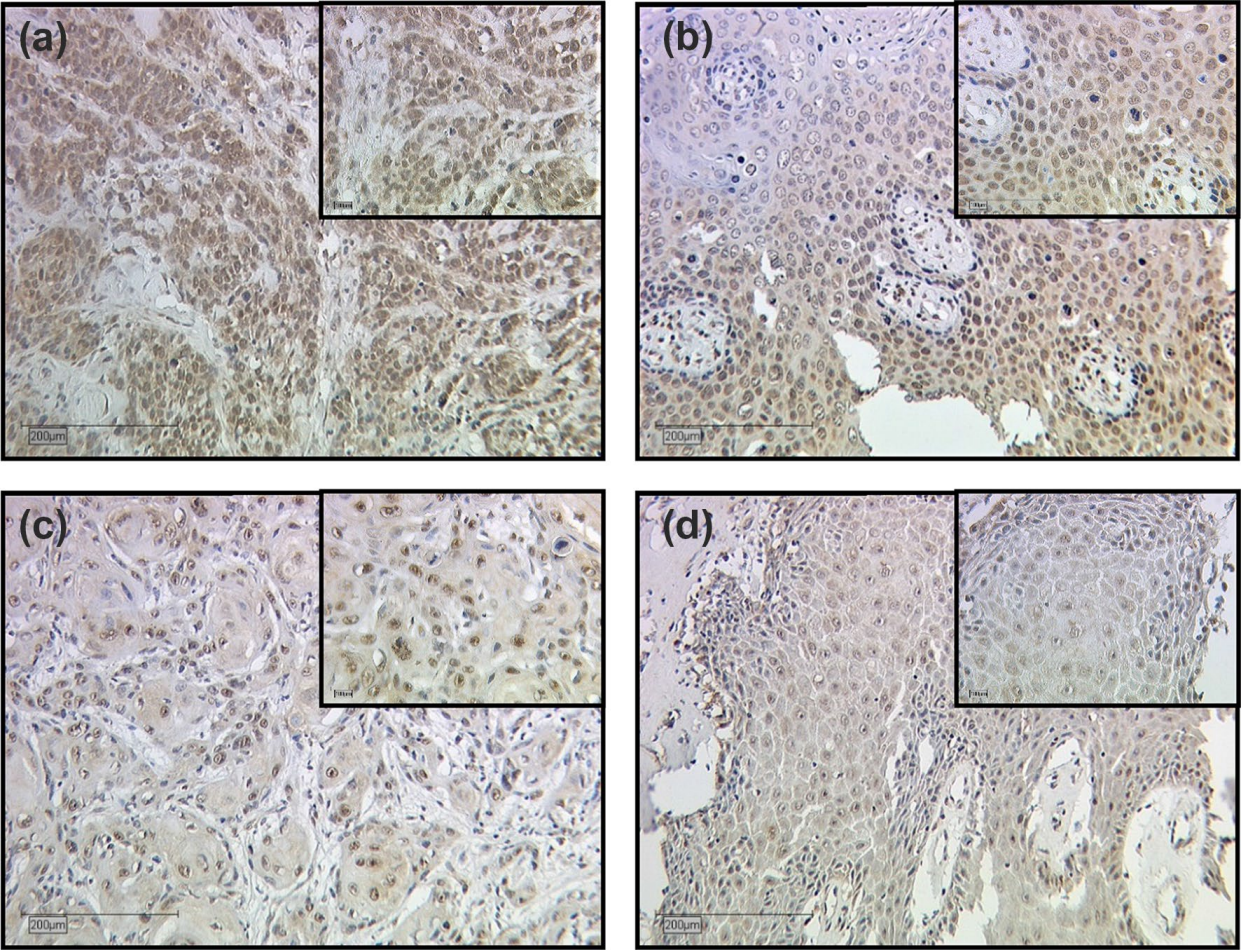
Expression of MTA1 in different histologic tumor subtypes of vulvar carcinoma. Representative photomicrographs are presented (10 × and 25 × magnification): **a** non-keratinizing vulvar carcinoma; **b** vulvar carcinoma of the condylomatous type; **c** keratinizing vulvar carcinoma; **d** verrucous vulvar carcinoma

**Fig. 4:**
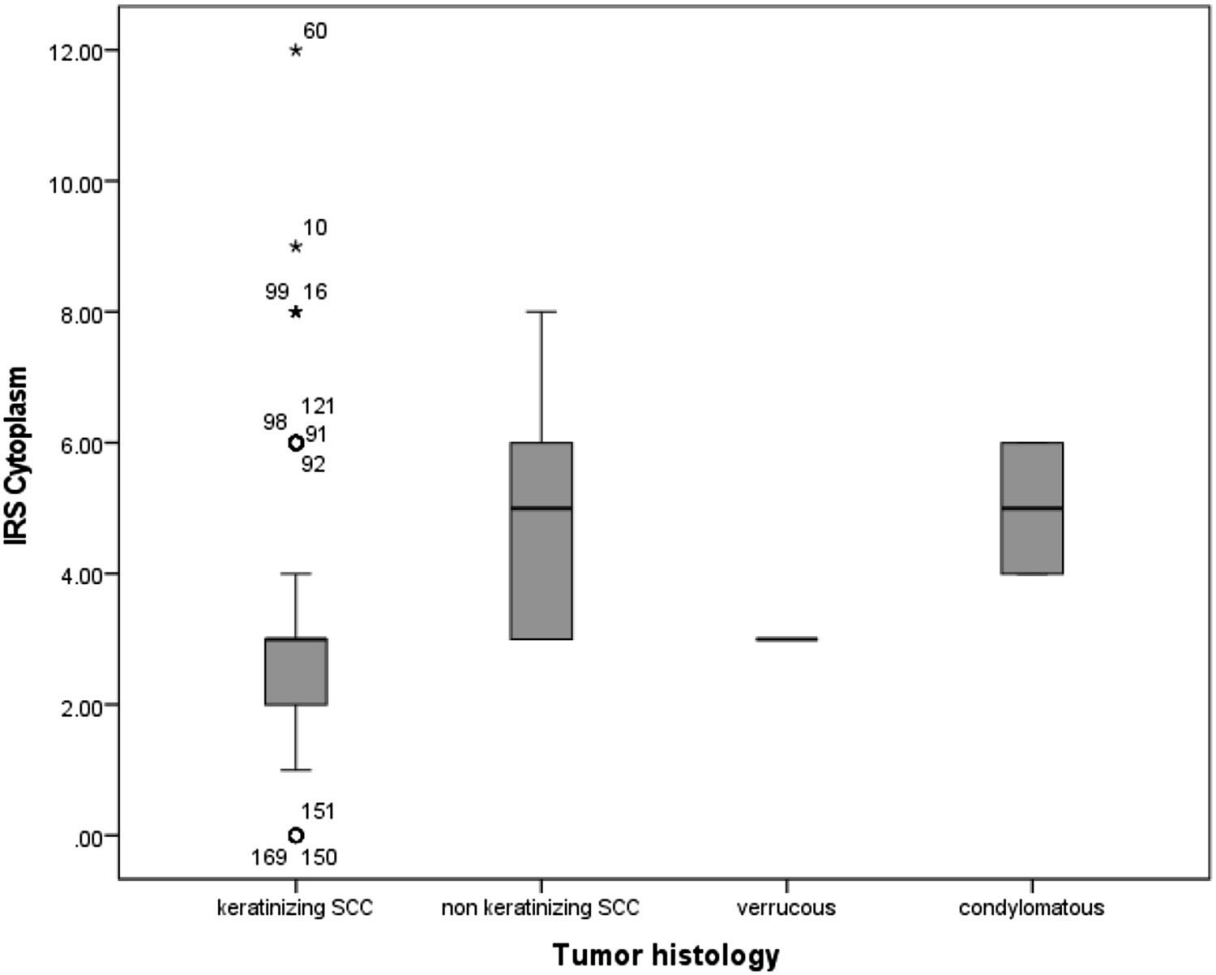
MTA1 expression in relation to tumor histology: MTA1 expression was significantly increased in non-keratinizing squamous cell carcinoma and in vulvar carcinoma of the condylomatous type (*p* = 0.038)

MTA1 expression differed depending on the tumor histology (Fig. 3). In non-keratinizing squamous cell carcinoma and in vulvar carcinoma of the condylomatous type, the expression of MTA1 in the cytoplasm was significantly increased compared to the other two tumor subtypes (*p* = 0.038, Fig. 4).

### Correlation of MTA1 positive staining with clinicopathologic parameters of the study group

#### Tumor size

**Fig. 5:**
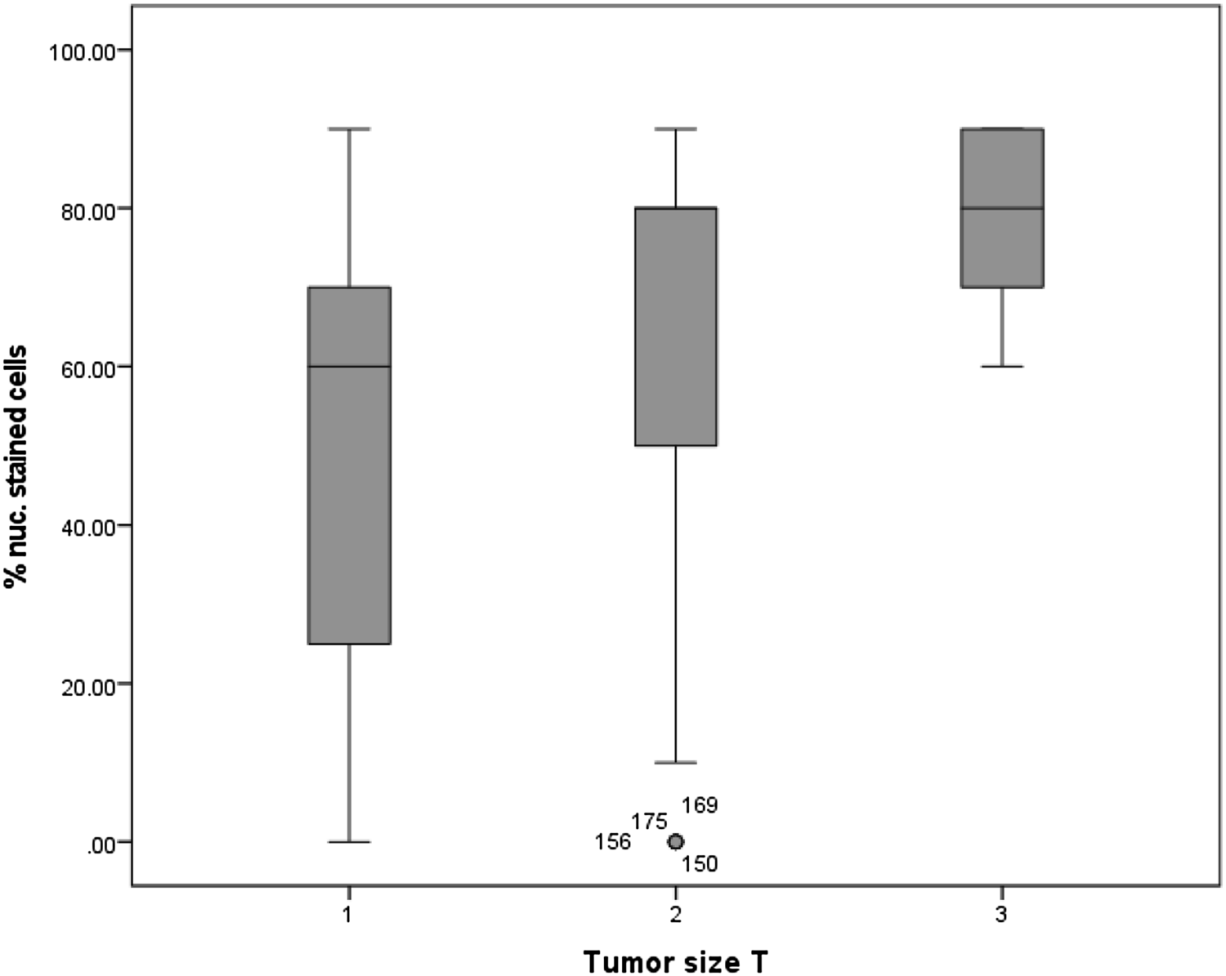
Percentage of cells with nuclear expression of MTA1 in relation to the tumor size T: percentage of cells expressing MTA1 was significantly increased in T2 and T3 carcinomas (*p* = 0.0001)

When classified by the tumor size, presented by the letter T in the TNM classification, a significant upregulation of MTA1 expression in vulvar cancer at higher cancer stages was observed (*p* = 0.0001). T1 carcinomas had a mean of 60% nuclear-stained cells, while in T2 and T3 carcinomas the mean percentage of stained cells was 80% (Fig. 5**)**. In addition, a significant correlation between MTA1 expression in the cytoplasm and tumors size was shown (*p* = 0.008).

#### FIGO score

**Fig. 6:**
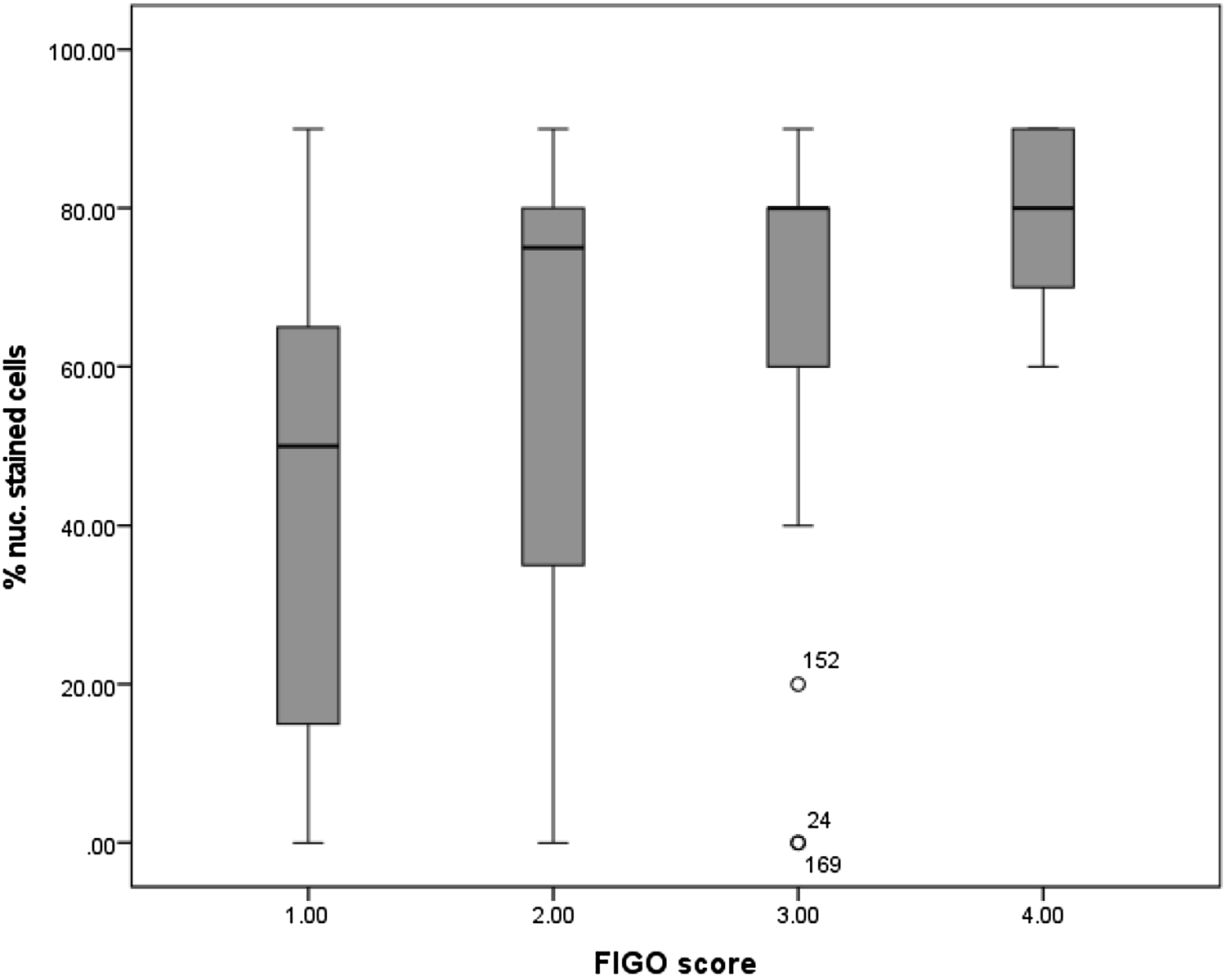
Percentage of cells with nuclear MTA1 expression in relation to FIGO stage: percentage of cells expressing MTA1 was significantly increased at higher FIGO stages (*p* = 0.001)

Similar results were obtained when the examined cancer tissues were classified by the FIGO staging system. The expression of MTA1 significantly increased with higher FIGO grading (*p* = 0.001). Carcinomas classified as FIGO 1 showed a lower percentage of stained cells than higher FIGO stages. Carcinomas graded as FIGO 2 showed a mean of 75% stained cells, whereas in FIGO 3 and FIGO 4 carcinomas, the mean percentage of stained cells was 80% (Fig. 6).

#### P16 status

**Fig. 7:**
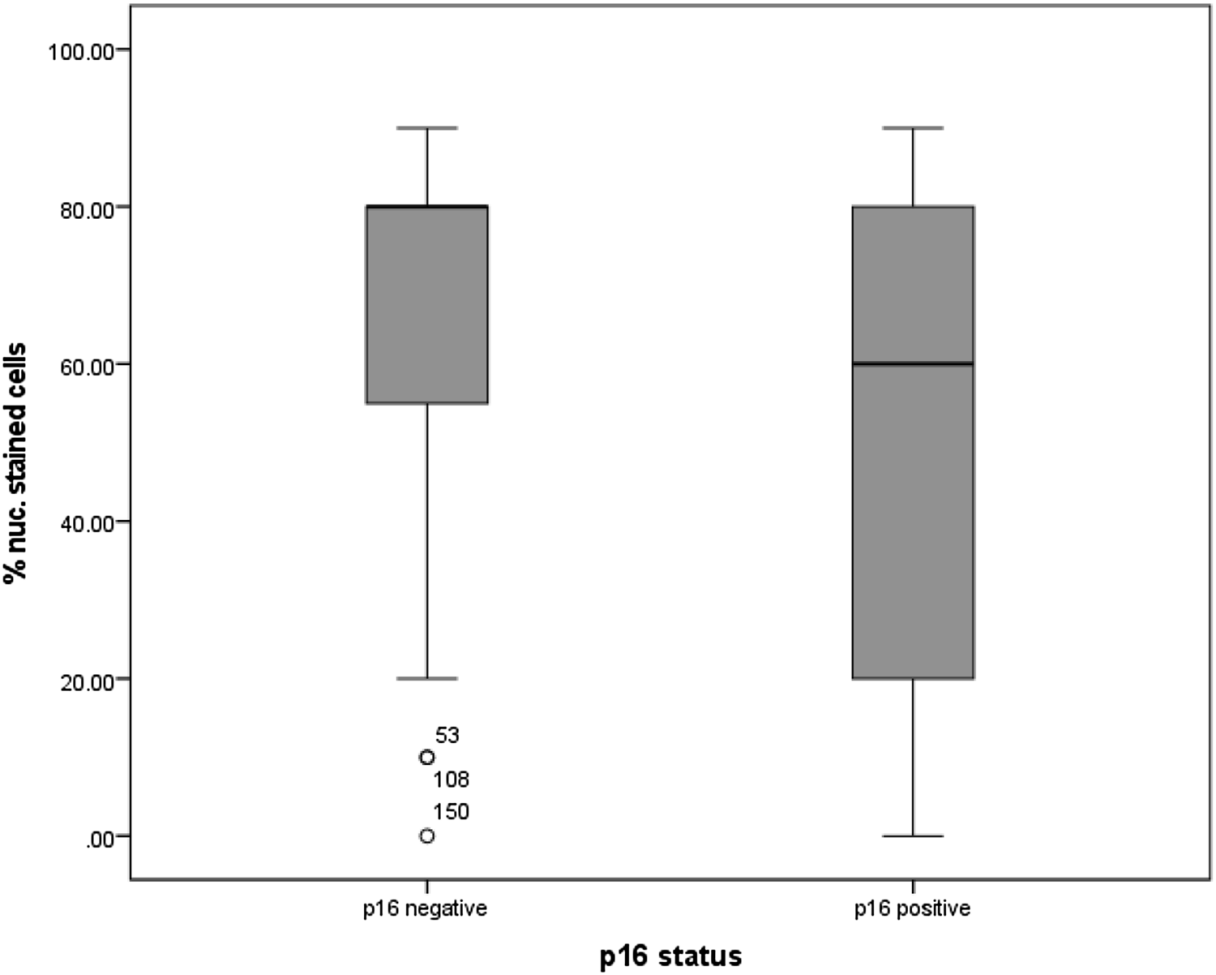
Percentage of cells with nuclear expression of MTA1 in relation to p16 status: percentage of cells expressing MTA1 was significantly higher in carcinomas with negative p16 status (*p* = 0.014)

Regarding the p16 status, used as a surrogate parameter for infection with human papilloma virus, the statistical analysis showed that the percentage of cells expressing MTA1 was higher in carcinomas with negative p16 status (*p* = 0.014, Fig. 7). In contrast, tumor slides with positive p16 status presented a lower percentage of nuclear staining. The other nuclear staining parameters showed also a significant result in the statistical analysis (intensity *p* = 0.031, percentage of positive cells *p* = 0.014 and IRS (*p* = 0.032).

#### Disease free survival

**Fig. 8:**
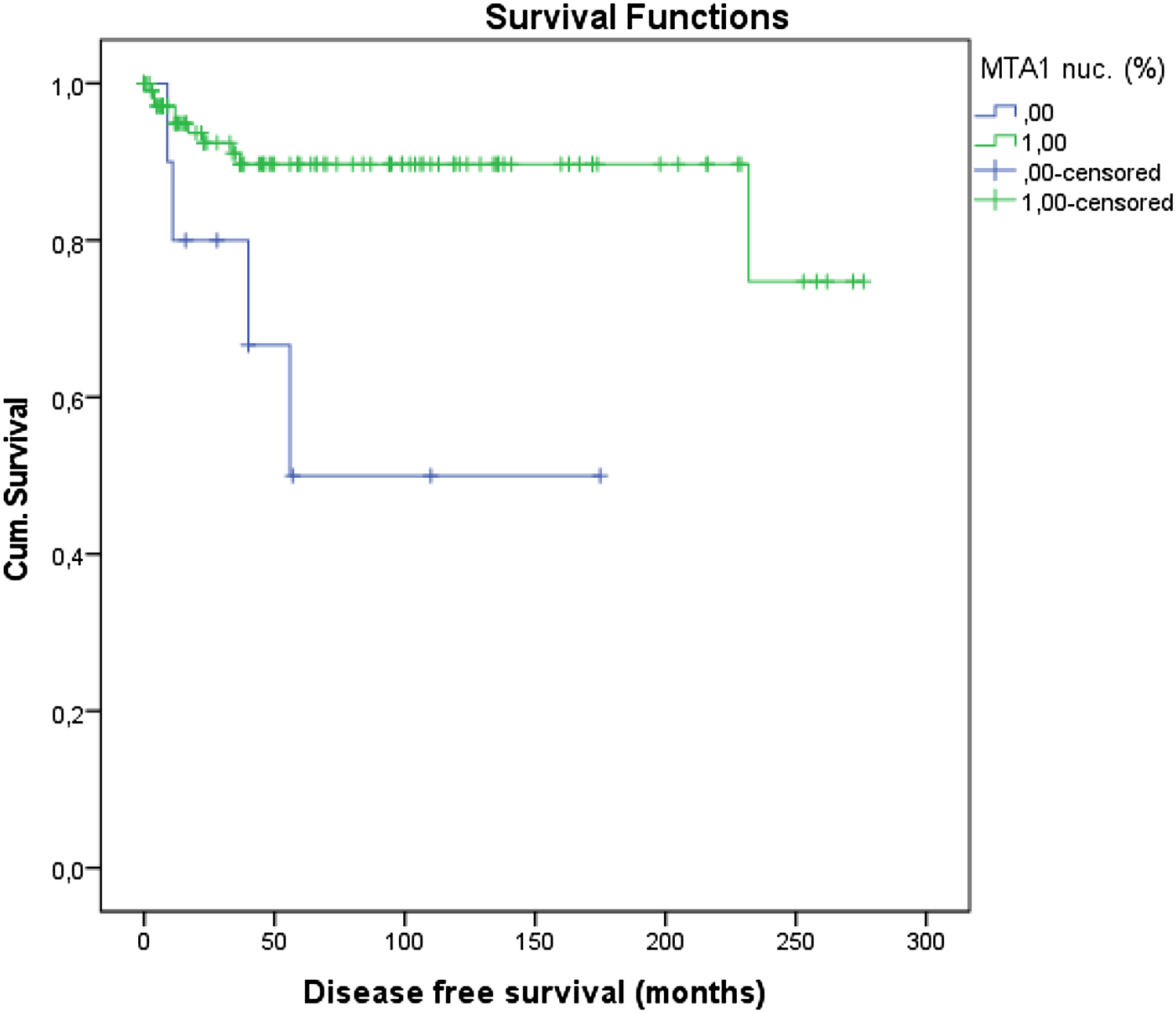
Kaplan–Meier survival curve: higher disease-free survival in patients with nuclear expression of MTA1 (*p* = 0.004)

According to the Kaplan–Meier survival curves analysis a significantly higher disease-free survival was shown in tumor samples with nuclear expression of MTA1 (*p* = 0.004, Fig. 8). No significant correlation was found between cytoplasmic expression of MTA1 and disease-free survival.

## Discussion

In the present study, we investigated the expression of MTA1 in vulvar cancer tissue and its correlation to above mentioned clinicopathological parameters of the study group. MTA1 was expressed both in the nucleus and in the cytoplasm in more than 90% of the examined vulvar carcinoma tissue sections, while there was no expression in normal vulvar tissue. The mean IRS value for both cell compartments was 3.3, corresponding to moderately strong staining, and indicating a similar distribution of MTA1 in the two cell compartments. This is also reflected in the correlation analysis, showing a clear positive correlation between the expression of MTA1 in the nucleus and the cytoplasm. MTA1 was originally thought to be a predominantly nuclear-localized protein associated with metastasis and negative prognosis (Toh et al. 1994). It is now known to be expressed equally in both the nuclear and the cytoplasmatic compartment (Liu et al. 2014). The subcellular localization of a protein is closely related to its function on a molecular level, implying a functional difference between nuclear and cytoplasmatic expressed MTA1. In fact, however, MTA1 appears to function as an oncogene both in the nucleus and in the cytoplasm and to regulate the activity of its target proteins via interaction with histone deacetylase 1 (HDAC1) and 2 (HDAC2) as a part of the NuRD complex.

Four histological tumor subtypes of vulvar carcinoma were included in this study and examined regarding their expression of MTA1. Non-keratinizing squamous cell carcinoma and vulvar carcinoma of the condylomatous type showed an increased expression of MTA1 in the cytoplasm compared to keratinizing squamous cell carcinoma and vulvar carcinoma of the verrucous type. Non-keratinizing squamous cell carcinoma and vulvar carcinoma of the condylomatous type are considered histological tumor types that are more frequent in younger women and mainly HPV-associated (Hoang et al. 2016). HPV-associated vulvar carcinomas have a better prognosis than HPV-independent tumor morphologies as keratinizing squamous cell carcinoma (McAlpine et al. 2017; Allo et al. 2019). In the case of vulvar carcinomas of the verrucous type, however, the association with HPV is intensively discussed. Several studies report an association with HPV 6 and 11, while others find no association with HPV (Nascimento et al. 2004; de Koning et al. 2008). The increased expression of MTA1 in the HPV-associated tumor morphologies, which are known to have a better prognosis, is contradictory to the fact that MTA1 appears to be a negative prognostic marker. However, it must be noted that a considerable number of HPV-dependent vulvar carcinomas show keratinization and therefore show similarities to HPV-independent tumors (McAlpine et al. 2017). Thus, a strict division into HPV-dependent and HPV-independent tumor morphologies is not possible purely by histological examinations. Another limiting factor is the low number of cases of vulvar carcinomas of the condylomatous (*n* = 2) and verrucous type (*n* = 3) in our patient population, which leads to a bias in the results. In our analyses, the expression of MTA1 was increased in non-keratinized squamous cell carcinoma and vulvar carcinoma of the condylomatous type, although these two tumor subtypes were not simultaneously associated with a worse prognosis in our patient population.

We found a significant upregulation of MTA1 expression in vulvar carcinoma at higher cancer grading as well as higher FIGO stages. With higher tumor size, a significant increase in expression of MTA1 in the nucleus as well as the cytoplasm was shown. T1-rated carcinomas had a mean percentage of 60% nuclear-stained cells, while T2 and T3 carcinomas had a mean percentage of 80% stained cells. Similar result was found regarding the FIGO grading. The higher the FIGO stage, the greater the percentage of nuclear-stained cells. Both parameters, the tumor stage of the TNM classification and the FIGO grading are used for the description of the extension of a tumor, not least to make prognostic statements. High stages are associated with a poorer prognosis for the patients. For ovarian (McAlpine et al. 2017) and cervical (Bruning et al. 2014) carcinoma, similar studies showed increased expression of MTA1 in aggressive tumors characterised by a high tumor stage. Therefore, our results support that MTA1 represents a significant factor in tumor progression in vulvar carcinoma and is higher expressed in advanced tumor stages.

Furthermore, the p16 status of the patients was examined in relation to the expression of MTA1. Since the HPV status of a lesion cannot be determined with sufficient certainty by haematoxylin–eosin (HE) staining alone, immunohistochemical staining of p16 is part of the clinical routine to make statements about an HPV infection. The overexpression of p16 is a direct consequence of cell cycle alteration that is induced by HPV oncogenes (Sano et al. 1998). Therefore, positive p16 staining is considered a surrogate parameter for HPV infection not only in vulvar cancer (Santos et al. 2006). Statistical analysis showed that with positive p16 status, the percentage of cells expressing MTA1 in the nucleus was lower than with negative p16 status. Conversely, this means that HPV-independent vulvar carcinomas express more MTA1 than HPV-dependent vulvar carcinomas. As mentioned above, HPV-independent vulvar carcinomas are associated with a worse prognosis (Allo et al. 2019). A specific reason for this is not apparent, as HPV-dependent and HPV-independent vulvar carcinomas differ significantly in their etiology, the average patient age at diagnosis, risk factors, molecular processes during pathogenesis as well as the precancerous lesions (Hoang et al. 2016). A more detailed investigation of MTA1 and its relation to HPV status in vulvar carcinomas will be the subject of future research. The analyses of the present study clearly show that HPV-independent vulvar carcinomas are characterized by increased MTA1 expression levels, which in turn underlines the importance of MTA1 as a negative prognostic marker for vulvar carcinoma.

For the lymph node status as a further parameter of the TNM classification and the grading, there were no significant correlations regarding the expression of MTA1. Likewise, there was no significant difference between the presence of metastases and MTA1 expression. However, it must be noted that the metastasis status was only documented in 8 of 177 cases. An investigation of this variable with a larger number of cases would have been very interesting since MTA1 has already been associated with metastasis and invasion in many different tumors and therefore bears the name “metastasis-associated” (Toh et al. 1999; Balasenthil et al. 2006; Kawasaki et al. 2008).

Regarding survival, a higher recurrence-free survival time was shown in tumor samples with nuclear expression of MTA1. Consequently, a reduced recurrence-free time can be assumed in cases without nuclear expression of MTA1. The antibody used in this study recognizes an epitope in the region of the C-terminus of the corresponding protein. This region is known for tumor-specific mutation (Mishra et al. 2004; Singh et al. 2006). Analogous to the immunohistochemical staining of p53 established in oncology, it is assumed that the staining is absent due to a mutation. This means that a mutation leads to a spliced-out C-terminus. Patients with the corresponding mutation could have an earlier recurrence. Similar results were found in hepatitis B virus (HBV)-associated hepatocellular carcinoma. The analysis of MTA1 and its exon 4-excluded form (MTA1dE4), the most abundant spliced variant of MTA1, showed that MTA1dE4 overexpression in tumor, but not MTA1, was associated with early recurrence. Li et al. concluded that expression is correlated with more aggressive tumor characteristics. At this point, this is also conceivable for vulvar carcinoma.

The expression of MTA1 seems to play an important role in the progression of vulvar cancer: advanced vulvar carcinomas are characterized by an increased expression of MTA1. A further examination of a possible downregulation or elimination of MTA1 would represent an interesting therapeutic approach in vulvar cancer.

## Data Availability

The data presented in this study are available on request from the corresponding author. The data are not publicly available due to ethical issues.
